# The “Chik Sign” in Neonatal Chikungunya

**DOI:** 10.1590/0037-8682-0157-2020

**Published:** 2020-06-12

**Authors:** Jayasree Chandramathi, Ashwin Prabhu, Anil Kumar

**Affiliations:** 1 Amrita Institute of Medical Sciences, Amrita Vishwa Vidyapeetham, Ponekara, Kochi, Kerala India.

**FIGURE 1:**
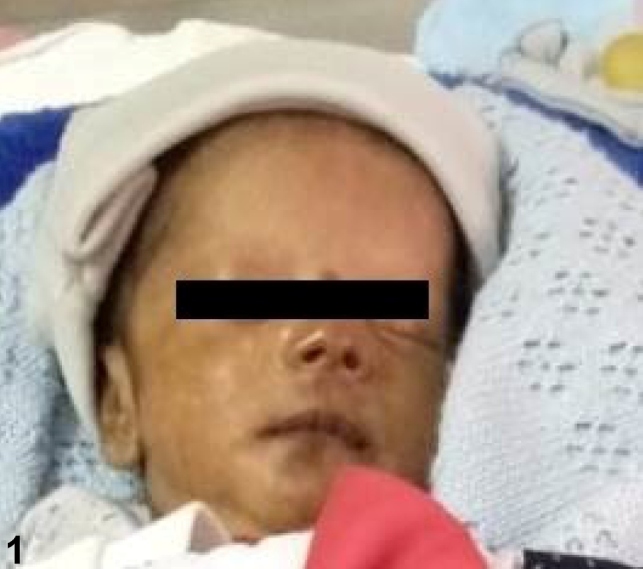
Brownish pigmentation of the nose, mouth and, cheeks.

**FIGURE 2:**
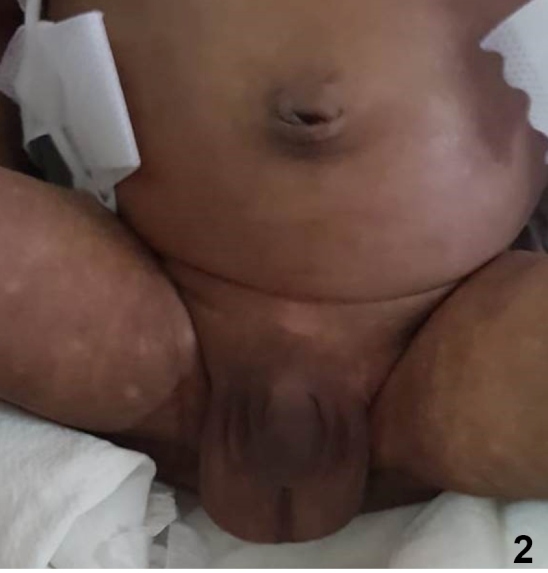
Brownish pigmentation of the genital area, groin and, thighs.

A 15-day-old male infant was referred to our hospital with a history of recurrent apnea, suspected sepsis, neonatal hyperbilirubinemia, and thrombocytopenia. The infant was ventilated at the referring hospital for recurrent apnea and was treated with intravenous antibiotics and other supportive measures. General examination at admission revealed marked hyperpigmentation of the face (Figure 1) and genital region (Figure 2). Systemic examination was unremarkable. The possibility of neonatal chikungunya was considered due to classical hyperpigmentation, clinical presentation, and thrombocytopenia. The patient’s mother reported having suffered a fever during the week preceding his delivery. The diagnosis in the neonate was confirmed by positive IgM antibodies to chikungunya. The child was treated symptomatically, recovered gradually, and was extubated on the third day of admission. Platelet count also normalized and the infant was discharged on the tenth day of admission. Transmission of chikungunya from mother to fetus is most likely when the mother is viremic at delivery. Though neurological, ocular, renal, and hematological manifestations have been described, the striking pigmentation of the nose, described as the “chik sign,” is the most recognizable feature in the diagnosis of neonatal chikungunya^1^ ^,^ ^2^. Differential diagnoses include congenital lupus, drug rash (due to imipenem, for instance), and bacterial infections (*Listeria monocytogenes*, *Staphylococcus epidermidis*), fungi (*Candida*), and viruses (human herpesvirus 6, enteroviruses)^1^. Knowledge of the “chik sign” as a cutaneous feature of chikungunya could be useful in resource-poor settings to detect unrecognized outbreaks of this arboviral fever, especially when the facilities for serological confirmation are not available.
